# Survival and Development of *Striacosta albicosta* (Smith) (Lepidoptera: Noctuidae) Immature Stages on Dry Beans, non-*Bt*, Cry1F, and Vip3A Maize

**DOI:** 10.3390/insects10100343

**Published:** 2019-10-13

**Authors:** Débora G. Montezano, Thomas E. Hunt, Alexandre Specht, Priscila M. C. Luz, Julie A. Peterson

**Affiliations:** 1 Department of Entomology, University of Nebraska-Lincoln, Lincoln, NE 68583, USA; 2 Department of Entomology, University of Nebraska-Lincoln, Haskell Agricultural Laboratory, Concord, NE 68728, USA; thunt2@unl.edu; 3 Embrapa Cerrados, Planaltina, 73310-970 Distrito Federal, Brazil; alexandre.specht@embrapa.br; 4 Department of Entomology, University of Nebraska-Lincoln, West Central Research & Extension Center, North Platte, NE 69101, USA; pricolomboluz@gmail.com (P.M.C.L.); julie.peterson@unl.edu (J.A.P.)

**Keywords:** *Bt* crops, transgenic corn, insect resistance management, integrated pest management, western bean cutworm

## Abstract

*Striacosta albicosta* is a crop pest that causes economic damage in the United States and Canada. Only maize and dry beans are shown to be suitable hosts, since larval development is incomplete on other hosts. The objective of this study was to describe the developmental parameters of immature stages of *S. albicosta* feeding on dry beans, non-*Bt*, Cry1F, and Vip3A maize. For Vip3A, mortality was 100% after 24 h. Larvae feeding on non-*Bt* maize had the highest larval survival (70.6%) compared to the other hosts. Maize expressing Cry1F had higher survival (31.3%) than dry beans (26.0%). Larvae feeding on dry beans had a significantly faster total development time (74.8 days), compared to 92.5 days for non-*Bt* and 96.2 days for Cry1F. All larvae developed through seven instars. Pupae from larvae that had fed on non-*Bt* maize were significantly heavier than pupae from other hosts. An understanding of *S. albicosta* immature development on various host plants is needed to improve recommendations for effective scouting, treatment timing, and economic thresholds. Differential development can result in an extended adult emergence period, and possibly result in assortative mating between *Bt* susceptible and resistant populations, which violates the assumption of random mating necessary for current resistance management strategies for *Bt* maize. Therefore, understanding the impact of host plant and transgenic traits on aspects of pest biology will aid in developing effective integrated pest management and insect resistance management strategies for this pest.

## 1. Introduction

*Striacosta albicosta* (Smith 1887) (Lepidoptera: Noctuidae), or western bean cutworm, is an endemic owlet moth pest in the North American corn belt. The status of *S. albicosta* as a pest of dry beans, *Phaseolus vulgaris* L., was reported for the first time in 1915 in Colorado [1,2], and then in 1950 in Nebraska [3]. Thirty-five years after its first report in dry beans, *S. albicosta* was reported damaging maize in Colorado and Nebraska [3,4]. Reports of host plants other than dry beans and maize are only on tomato, *Solanum lycopersicum* L., ground cherry, *Physalis* spp., and black nightshade, *Solanum nigrum* L. [5,6]. However, according to [6] and via reports of field damage, maize and dry beans are the only suitable hosts allowing for complete development. Starting in 1999, changes in cultural practices, such as reduced tillage, reduced use of broad-spectrum insecticides following the adoption of *Bt* maize, pest attributes such as long-distance dispersal ability, and release from competition with other ear-feeding Lepidoptera, among other possible factors [7,8], enabled this pest to expand its distribution eastward to Pennsylvania, New York, and Quebec, Canada [8,9,10,11]. In addition, there have been recent reports of damage to maize and dry beans in Mexico [12].

On dry beans, early instars of *S. albicosta* will feed primarily on leaves and flower buds, and later instars will feed on young bean pods until they become prepupae [3,13]. Similar behavior is observed on maize, where early instars will feed on reproductive tissue such as tassels and pollen during early instars, and later development will occur in the ear on kernels [14]. Reports of yield loss in maize vary, ranging from 248.83 kg/ha [15] to 945.52 kg/ha [16]. The most common management practices to control *S. albicosta* are the use of insecticides and the planting of genetically engineered maize that produces insecticidal proteins from the bacterium *Bacillus thuringiensis* (*Bt*) [1,3,17].

When first released, transgenic maize event TC1507 (co-developed by Dow AgroSciences and Pioneer Hi-Bred International, Johnston, IA) expressing Cry1F protein provided approximately 80% control of *S. albicosta* [18], but recent studies report reduced control of *S. albicosta* with Cry1F-expressing maize hybrids [19,20]. Another tactic used to control *S. albicosta* is maize expressing vegetative insecticidal protein Vip3A, which is highly efficacious [21]. This protein has shown no loss of efficacy for *S. albicosta*; however, it is not yet as widely planted as Cry1F in regions such as Nebraska [17], were *S. albicosta* is a significant pest.

Several studies show that sublethal effects for Noctuidae feeding on *Bt* crops are very common and present a variety of consequences, such as prolonged larval development, reduced pupal weight and size, reduced fecundity, and delayed emergence [22,23,24]. Furthermore, [25] observed that delayed development of resistant *Pectinophora gossypiella* L. adults resulted in assortative mating among *Bt* resistant and susceptible individuals. Consequences of sublethal effects may have important implications for *S. albicosta* population dynamics [24] and could violate a core assumption of the high-dose/refuge strategy in insect resistance management (IRM) for *Bt* crops [26]. A requirement for the high-dose/refuge strategy is that target pests have a refuge from *Bt* toxins to maintain a source of *Bt* susceptible alleles to decrease selection for resistance. It is expected that resistance is recessive, and *Bt* susceptible individuals from the refuge will mate with rare resistant individuals, reducing the probability that resistant insects will mate with one another, and consequently reduce the frequency of subsequent resistant individuals [27,28]. Because host plants directly affect the growth and development of insects, understanding the effect of host plants on the larval development and survival of insect pests is necessary for developing effective IRM strategies.

Considering that *S. albicosta* is now one of the most important maize and dry beans pests in the U.S. corn belt and regions of Canada, the objective of this study was to determine the effect of dry beans and maize (non-*Bt*, Cry1F, and Vip3A) on the development and survival of immature stages of *S. albicosta*, to improve resistance management and integrated pest management (IPM) recommendations for this pest.

## 2. Material and Methods

### 2.1. Insects, Plants and Laboratory Conditions

Experiments were conducted under laboratory conditions at the Agroecosystems Entomology Laboratory located at the West Central Research & Extension Center in North Platte, NE, USA. Biology studies were conducted with neonate larvae from egg masses laid by field-collected moths, following methods by [29]. Collections were made in 2017 near Grand Island, Nebraska, USA (40° 54’ 43.10” N, 98° 16’ 33.60” W). In the laboratory, egg masses were placed on moistened filter paper inside Petri dishes and held at 26.6 ± 1 °C, 70–80% relative humidity (RH) and 16:8 h (L: D), and monitored daily for hatching. Maize expressing Cry1F (SmartStax, DKC 61-55 RIB, DeKalb), Vip3A (Agrisure Viptera, G99z33-311A, Syngenta), or no *Bt* proteins (DKC 61-52, DeKalb, from the same hybrid family as the Cry1F plants) were grown under identical field conditions and standard agronomic practices for the region, with no insecticide applications, at the University of Nebraska-Lincoln West Central Research and Extension Center in North Platte, Nebraska, USA (41°05′23.60″ N, 100°46′20.40″ W). Dry beans (Great Value Pinto Beans, Walmart, Inc.) were grown in a germination mix in four-liter containers under greenhouse conditions (27.7 ± 2 °C, 70–80% RH, and 16:8 h (L:D)). Plant tissue was harvested daily in order to provide fresh tissue for larvae throughout the experiment. Cry1F and Vip3A expression was confirmed using ImmunoStrip^®^ for Vip3A (Agdia, Inc. STX 83500/0050) and ImmunoStrip^®^ for *Bt*-Cry1F (Agdia, Inc. STX 10900/0050). All experiments were performed in a rearing room (26.6 ± 1 °C, 70–80% RH) with evaluations performed daily at 10:00 a.m. Central Daylight Time.

### 2.2. Larval Stage

The larval stage was divided into two periods as described in [29]. An “active larval period” where larvae were active and feeding, and an “inactive larval period” (prepupal period) at the end of the larval developmental stage, which is characterized by the interruption of feeding, a decrease in size due to loss of body water, fading of coloration, and the construction of the prepupal chamber using body fluids and humidity to cement organic materials available in the container.

### 2.3. Active Larval Stage

The experiment was initiated with 800 neonates within two hours of hatching. Larvae were reared individually following [30] on four different natural diets: Dry beans, non-*Bt* maize, *Bt* maize expressing Cry1F, and *Bt* maize expressing Vip3A (200 neonates were used for each diet). Neonates were selected from 10 different egg masses and were randomly assigned to diet treatments and placed in plastic containers (300 mL volume, 10 cm diameter, 15 cm height; ULINE, Wisconsin, U.S.). All insects were housed individually throughout the experiment. In order to maintain humidity, holes were punched in the container lids and a small wad of cotton (~1 cm in diameter) moistened with distilled water was added to each container. First, second and third instars were fed daily with a 3–4 g piece of fresh maize tassel from each different type of maize (non-*Bt*, Cry1F, and Vip3A) or 3–4 g of dry bean leaves, depending on the assigned diet treatment. 

Maize kernels (~100 g) plus silk (3–4 g) were added to the container when larvae reached the third instar. For dry beans, bean pods (~100 g) were added to the container after larvae molted to the third instar. The plant tissue and moist cotton were replaced daily. Daily observations were made to verify the survival and development of the larvae by collection of the molted head capsules. The head capsules were individually stored and labeled by larva in microcentrifuge tubes and the head capsule width was measured with a micrometer under a microscope. The mean growth ratio was calculated by dividing the mean head capsule width of each instar by the mean head capsule width of the previous instar.

### 2.4. Inactive Larval Period (Prepupal Period)

At the beginning of the inactive larval period (prepupal period), the insects were transferred into a clean transparent plastic container (300 mL volume, 10 cm diameter, 15 cm height) containing a mixture of autoclaved play sand (QUIKRETE^®^ Premium Play Sand^®^, #1113) and tap water. Daily observations were made to verify the survival and to record pupal metamorphosis.

### 2.5. Pupal Stage

Pupae were kept in the same container and conditions as in the prepupal period. Daily activities consisted of maintaining the moisture with ~10 mL of distilled water, and recording the emergence of adults. Sex identification was performed following [31] on the second day after pupation, when the cuticle is further hardened, and pupae were weighed on a semi-analytical balance (precision of 0.01 g). The identity of each larva was preserved throughout the study, so it was possible to track the development of each *S. albicosta* from neonate to pupa.

### 2.6. Data Analysis

Stage duration, size, and weight were subjected to the Shapiro–Wilk normality test. Means were subjected to analysis of variance (ANOVA) through the F test (*p* < 0.05). When statistically significant, the means were compared by Tukey’s test (*p* < 0.05) using the PROC GLIMMIX procedure in SAS (SAS software v. 9.4; SAS Institute Inc. Cary, NC, USA). Survivorship curves were elaborated using Kaplan–Meier estimator [32] and comparisons between survival curves were made using Log Rank test with R version 3.5.1. Statistical analyses were not possible for pupal duration due to high mortality at this stage.

## 3. Results

### 3.1. Overall Development

**Figure 1:**
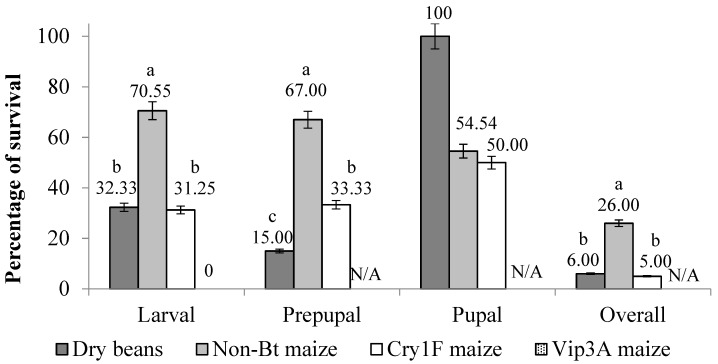
Survival (% ± SE) of *S. albicosta* immature stages within each stage, from larvae reared on dry beans, non-*Bt* maize, Cry1F maize, and Vip3A maize (100% mortality) under controlled conditions. Different letters indicate significant differences between diets within a developmental stage (*p* < 0.05). Means comparison based on Tukey’s test. Statistical analyses were not possible for pupal duration due to high mortality.

**Figure 2:**
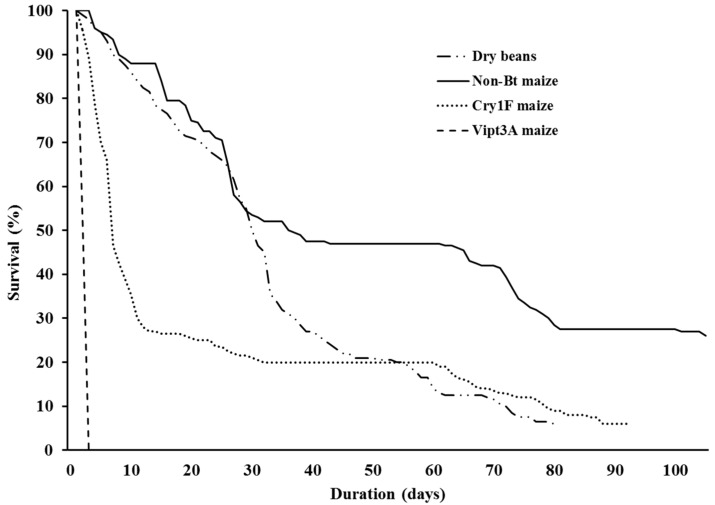
Survival curves of immature *S. albicosta* (larvae, prepupae, and pupae) from larvae reared on dry beans, non-*Bt*, Cry 1F and Vip3A maize under controlled conditions. Note that only the Vip3A maize curve reached zero, and the others stopped at the percentage of adults that emerged (Log Rank test, χ^2^ = 913.00, df = 3, *p* < 0.001).

The overall survival of *S. albicosta* immature stages (larvae, prepupae, and pupae) reared under controlled conditions was significantly higher on non-*Bt* maize compared to dry beans and Cry1F maize (F = 34,82; df = 120; *p* < 0.0001) (Figure 1 and Figure 2). There were no significant differences for overall survival between larvae fed dry beans and Cry1F (*p* = 0.3586) (Figure 1); however, comparisons among survivorship curves showed significant differences between all diets (Figure 2, Table 1). After 48-hour exposure to Vip3A maize, all *S. albicosta* larvae had died (Figure 2).

**Figure 3:**
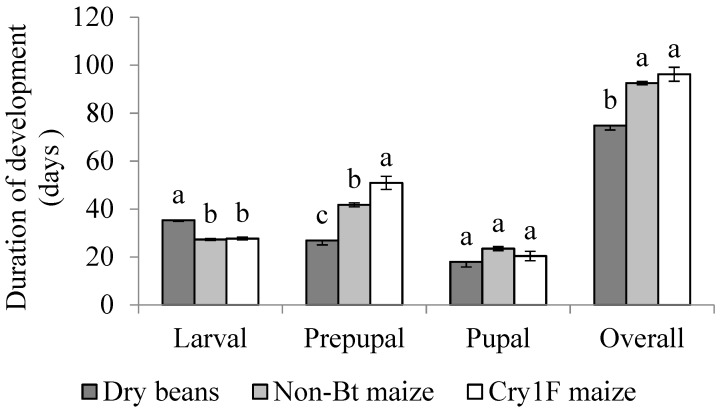
Mean larval duration (days ± SE) of *S. albicosta*, during each immature stage, from larvae reared on dry beans, non-*Bt* maize, and Cry1F maize under controlled conditions. Different letters indicate significant differences between diets within a developmental stage (*p* < 0.05). Means comparison based on Tukey’s test.

The length of complete immature stage development, including the active larval period, inactive larval period (prepupal period), and pupal stage, was significantly different among diets (F = 17.95; df = 70; *p* < 0.0001) (Figure 3). In overall, the fastest development was for larvae fed on dry beans (74.83 ± 1.88 days) compared to both non-*Bt* (92.53 ± 0.68 days) and Cry1F maize (96.20 ± 4.97 days) (*p* < 0.0001) (Figure 3). There was no difference in overall development when comparing non-*Bt* maize and Cry1F maize (*p* = 0.0648) (Figure 3). Comparison of survivorship curves showed significant differences between all diets when including all immature stages of development (Figure 2, Table 1).

### 3.2. Active Larval Period

In the active larval period, the lowest survival after Vip3A (0%) was observed for larvae that were fed Cry1F maize (31.25%), followed by larvae fed dry beans (32.33%), with the highest survival observed on non-*Bt* maize (70.55%). Survival during the active period for larvae fed non-*Bt* maize was significantly higher when compared to dry beans and Cry1F maize (F = 43.28; df = 120; *p* < 0.0001), and there was no difference between dry beans and Cry1F maize (*p* = 0.0758) (Figure 1). When looking at the duration of the active larval period, there was a significant difference among diets (F = 34.82; df = 120; *p* < 0.0001). Larvae fed dry beans had the longest active larval period (35.33 ± 0.42) compared to non-*Bt* maize (27.29 ± 0.43) and Cry1F maize (27.69 ± 0.58) (*p* < 0.0001), and there was no difference among larvae fed non-*Bt* and Cry1F maize (Figure 3).

**Figure 4:**
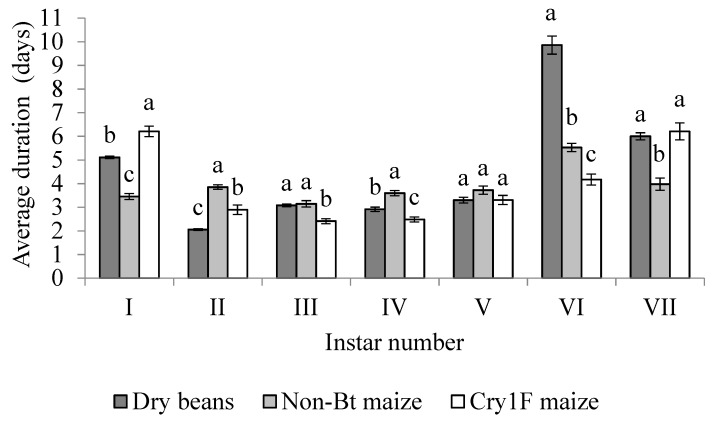
Mean duration (days ± SE) of *S. albicosta* active feeding larvae, during each instar, from larvae reared on dry beans, non-*Bt* maize, and Cry1F maize, under controlled conditions. Different letters indicate significant differences between diets within an instar (*p* < 0.05). Comparison of means based on Tukey’s test.

All larvae developed through seven instars. The duration of each instar was variable based on diet. All instars had significant differences between diets with the exception of the fifth instar (F = 2.18; df = 119; *p* = 0.12) (Figure 4). The longest development time was for larvae fed dry beans during the sixth instar (Figure 4).

With respect to the size of the head capsule, larvae fed dry beans had larger sized head capsules for the first six instars when compared to larvae fed non-*Bt* or Cry1F maize, with significant differences for second, third, and fourth instars (Table 2). However, the seventh instar head capsule was significantly smaller for larvae fed dry beans, when compared to the other diets, resulting in a lower growth ratio (Table 2).

### 3.3. Inactive Larval Period (Prepupal Period)

In the inactive larval period (prepupae), survival was 15.00% on dry beans, 67.00% on non-*Bt* maize, and 33.33% on Cry1F maize, with a significant difference for non-*Bt* maize compared to dry beans and Cry1F maize, and between dry beans and Cry1F maize (*p* < 0.0001) (Figure 1). There was a significant difference among diets for the total duration of the prepupal period (F = 45.95; df = 119; *p* < 0.0001). The prepupal period was significantly shorter for larvae fed dry beans (26.89 ± 1.85), followed by non-*Bt* maize (41.73 ± 0.84). The longest prepupal period was observed for larvae fed Cry1F maize (50.90 ± 2.73) (*p* < 0.001) (Figure 3).

### 3.4. Pupal Stage

**Figure 5:**
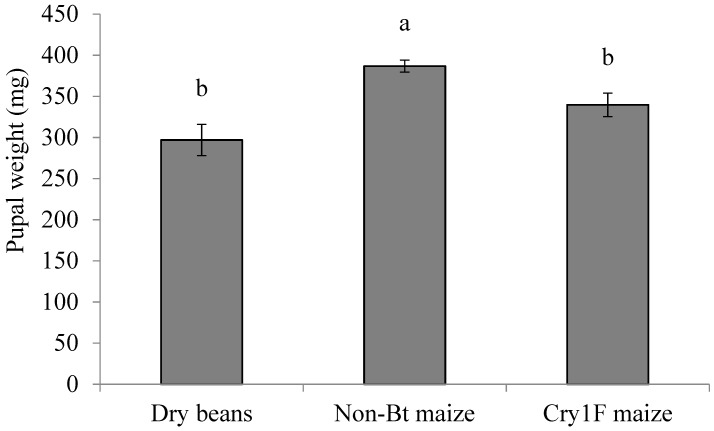
Pupal weight (mg ± SE) of *S. albicosta* larvae reared on dry beans, non-*Bt* maize, and Cry1F maize under controlled conditions. Different letters indicate significant differences between larval diets (*p* < 0.05). Means comparison based on Tukey’s test.

Pupal survival was 100.0%, 54.4% and 50.0% for larvae fed dry beans, non-*Bt* maize and Cry1F maize, respectively (Figure 1). The high mortality and low number of subjects in the prepupal period prevented statistical analysis. Pupal development lasted an average of 23.50 ± 8.08 days, and did not differ significantly among individuals that were fed dry beans, non-*Bt* or Cry1F maize (F = 2.96; df = 70; *p* = 0.060) (Figure 3). There were significant differences among average pupal weight (F = 45.95; df = 119; *p* < 0.0001) with respect to larval diet, with significantly heavier pupae for larvae fed non-*Bt* maize (*p* < 0.001). Larvae fed dry beans and Cry1F maize did not differ in pupal weight (*p* > 0.0005) (Figure 5). Due to low survival of the pupal stage, it was not possible to compare statistical differences between sexes.

## 4. Discussion

Our data show that different larval host plants significantly affected the performance of *S. albicosta* during immature development, including survival, duration and size. Overall, larvae fed non-*Bt* maize exhibited the highest survival, longest active larval stage, largest head capsule, and largest pupal size. High mortality and other developmental factors for the other maize hosts can be attributed to the sublethal effects of Cry1F and the lethal effect of Vip3A. This experiment tried to mimic actual field feeding behavior, offering suitable plant tissues for early and late instars. Nevertheless, high mobility and the capacity for choice of different plant tissues and hosts in nature can provide the larva with different nutritional compositions, likely more suitable for development, which cannot be reproduced under laboratory conditions. In addition, herbivory in the field may result in the upregulation of induced plant defenses, affecting larval development [33]. Therefore, considering these and other biotic and abiotic factors, the rates of survival and development could be different under field conditions.

The survival rates reported in this study for *S. albicosta* immatures fed their typical host plant were low (Figure 1 and Figure 2). This is similar to several laboratory and field studies that reported high mortality rates for immature stage *S. albicosta* [1,6,34,35,36], where highest mortality was observed primarily during the prepupal period. In this study, the prepupal period also had the lowest survival rate, similar to larvae fed artificial diet [29]. Low prepupal survival may be associated with important changes required for metamorphosis and it is possible that laboratory conditions did not meet these requirements. However, high prepupal mortality rates are also observed under field conditions [8,34], might be related to factors associated with intrinsic aspects of the species with respect to overwintering and the prepupal to pupal molting process [35,36]. *Striacosta albicosta* is univoltine, having an obligatory prepupal diapause, and therefore depends on various hormonal and external stimuli to complete its life cycle, strongly affecting survival under both field and laboratory conditions. Such stimuli have never been investigated for *S. albicosta*.

The number of instars reported in this study corroborates with data presented by [29], showing that larvae typically develop through seven instars. This result disagrees with previous reports, where *S. albicosta* has five or six instars [36,37]. In the present study under laboratory conditions, the collection and measurement of all head capsules allowed us to determine with precision that larvae developed through seven instars [29]. Larval development in Noctuidae is commonly through six or seven instars; however, several factors can influence the number of instars, including temperature, photoperiod, food quantity and quality, humidity, injury, inheritance, and sex [38,39,40]. Our studies were conducted under controlled laboratory conditions, and all larvae developed through the same number of instars for all tested diets. Our results do not agree with the compensation scenario hypothesis that additional instars are inserted under poor conditions when larvae fail to reach a species-specific threshold-size with the “normal” instar number [39,40,41].

The pupal weight observed in this study (Figure 5) was significantly higher for larvae fed on non-*Bt* maize than the other hosts, which was also higher than weights reported for larvae fed on artificial diet [29]. The lighter pupae developed from the larvae fed Cry1F *Bt* maize and dry beans may lead to smaller adults, and may lead to lower fecundity as reported for other Noctuidae species [42]. Lower fecundity can also decrease the expectation of successful offspring from random mating between *Bt* susceptible and resistant insects, which is an assumption of the high-dose/refuge strategy and an important consideration in developing resistance management plans.

Development of *S. albicosta* on Cry1F maize is particularly important because there are already reports of control failures and resistance to this toxin in *S. albicosta* [19,20], but there is no data showing the sublethal effects of this toxin on the development of *S. albicosta* immature stages. Our results indicate that in addition to reduced larval survival, *S. albicosta* takes on average four days longer to complete its active larval period on Cry1F maize compared to non-*Bt* maize, and 17 days longer compared to dry beans. These results are not uncommon for Noctuidae fed *Bt* toxins. Similar results were observed for *Helicoverpa zea* (Boddie) shortly after the introduction of Cry1Ab; 15–40% of *H. zea* larvae observed feeding on Cry1Ab survived, and survivors presented delayed development [22,23,42,43]. There was a six to seven-days delay in emergence for *H. zea* feeding on *Bt* maize compared to those on non-*Bt* maize under normal growing conditions [22]. Because prepupal survival and adult emergence was low in the present study, statistical analyses of adult emergence or adult longevity were not conducted. However, it is possible that a delay in immature stage development will result in delayed adult emergence [22,25]. If that is true, delayed development may result in asynchrony of emergence among resistant and susceptible adults, which has been observed for other pests feeding on *Bt* transgenic maize [22,23,25]. Developmental asynchrony would violate a main assumption of the high-dose/refuge strategy in IRM for *Bt* crops [24,26], which assumes that random mating will occur between susceptible and resistant insects. Although this is a possible scenario, the current study was conducted under laboratory conditions, so further research needs to be executed under field conditions to determine whether *S. albicosta* will exhibit asynchronous adult emergence.

Results from this study showed high efficacy of Vip3A in controlling neonates of *S. albicosta*, with 100% mortality within 24 h of feeding. However, an important consideration regarding the continued efficacy of Vip3A on *S. albicosta* is the combination of larval movement and instar-specific susceptibility in an integrated refuge scenario. Susceptibility to Vip3A is higher for *S. albicosta* neonates compared to third and fifth instar larvae [44]. Due to the ability of larvae to exhibit both inter- and intra-plant movement [14,45], they may grow and develop on non-*Bt* maize before they feed on Vip3A maize. An understanding of these interactions is crucial to design effective IRM strategies. *Striacosta albicosta* is becoming one of the most important maize pests in parts of the North American corn belt [8]. With recent control failures and the documented resistance of *S. albicosta* to Cry1F *Bt* maize, the only *Bt* toxin currently available that effectively controls this pest is Vip3A. Development of resistance by *S. albicosta* will be a significant threat to the sustainability of *Bt* crops. Thus, understanding the biological, ecological, and genetic factors, including life history, shifts in susceptibility, and sublethal effects of Vip3A, will be critical for the sustained use of this toxin.

## 5. Conclusions

Our results provide significant insight into how host plants, including *Bt* maize, affect *S. albicosta* immature developmental stages. The result that larvae developed faster on dry beans compared to maize is of particular importance in making treatment-timing decisions during pest management. Heavier pupae were obtained from non-*Bt* maize, which has important implications for resistance management if fitness of moths emerging from refuge and non-refuge areas is not equal. Our results also confirmed that the prepupal period is the most vulnerable to mortality, an important consideration in rearing this insect pest. Given the increasing importance of *S. albicosta* in maize production regions, our work lays the foundation for future studies evaluating the survival, population dynamics, and reproduction of this species on maize hybrids expressing *Bt* toxins, which will be critical for the design of effective IRM plans to sustain use of these traits.

## Figures and Tables

**Table 1:** Chi-square and *p*-values from Log Rank test for comparison between survival curves.

Survival Curves Comparisons	df	χ^2^	*p*
General comparison among all curves	3	913.00	<0.001
Dry beans x Cry1F maize	1	19.0	<0.001
Dry beans x Non-Bt maize	1	32.2	<0.001
Dry bean x Vip3A maize	1	422.0	<0.001
Cry1F maize x Non-Bt maize	1	88.8	<0.001
Cry1F maize x Vip3A maize	1	331.0	<0.001
Vip3A maize x Non-Bt maize	1	345.0	<0.001

**Table 2:** Width (mm) of head capsules of *S. albicosta* at each instar and respective growth ratios from larvae reared on dry beans, non-*Bt* maize, and Cry1F maize under controlled conditions. Sample sizes indicated by n represent those larvae that survived to pupation and whose head capsules were recovered at every instar.

	Dry Beans (*n* = 16)		Non-*Bt* (*n* = 34)		Cry1F (*n* = 30)	
Instar	Mean ± SE	Growth ratio	Mean ± SE	Growth ratio	Mean ± SE	Growth ratio
I	0.39 ± 0.01 a	---	0.37 ± 0.01 a	---	0.39 ± 0.00 a	---
II	0.62 ± 0.01 a	1.59	0.55 ± 0.01 b	1.49	0.56 ± 0.01 b	1.44
III	0.92 ± 0.02 a	1.48	0.88 ± 0.01 b	1.6	0.83 ± 0.02 b	1.48
IV	1.46 ± 0.03 a	1.59	1.32 ± 0.02 b	1.5	1.32 ± 0.04 b	1.60
V	2.12 ± 0.06 a	1.45	1.96 ± 0.02 a	1.48	2.00 ± 0.04 a	1.51
VI	2.92 ± 0.04 a	1.39	2.85 ± 0.01 a	1.45	2.93 ± 0.04 a	1.46
VII	3.33 ± 0.06 b	1.13	3.64 ± 0.02 a	1.28	3.66 ± 0.02 a	1.25
Mean	---	1.44	---	1.47	---	1.46

Different letters in a row indicates significant differences among diets within a developmental stage (*p* < 0.05). Comparison of means based on Tukey’s test.

## References

[1] Hoerner J.L. (1948). The cutworm *Loxagrotis albicosta* on beans. J. Econ. Entomol..

[2] McCampbell S.C. (1941). Cutworm control. Annual Report.

[3] Hagen A.F. (1962). The biology and control of the western bean cutworm in dent corn in Nebraska. J. Econ. Entomol..

[4] Douglass J.R., Ingram J.W., Gibson K.E., Peay W.E. (1957). The western bean cutworm as a pest of corn in Idaho. J. Econ. Entomol..

[5] Western Bean Cutworm and Its Control. http://agris.fao.org/agris-search/search.do?recordID=US201300613361.

[6] Blickenstaff C.C., Jolley P.M. (1982). Host plants of western bean cutworm. Environ. Entomol..

[7] Hutchison W.D., Hunt T.E., Hein G.L., Steffey K.L., Pilcher C.D., Rice M.E. (2011). Genetically engineered *Bt* corn and range expansion of the western bean cutworm (Lepidoptera: Noctuidae) in the United States: A response to Greenpeace Germany. J. Integr. Pest Manag..

[8] Smith J.L., Baute T.S., Sebright M.M., Schaafsma A.W., DiFonzo C.D. (2018). Establishment of *Striacosta albicosta* (Lepidoptera: Noctuidae) as a primary pest of corn in the Great Lakes region. J. Econ. Entomol..

[9] Baute T. (2009). Current distribution of western bean cutworm in the Great Lakes region. CropPest Ont. Newsl..

[10] Tooker J.F., Fleischer S.J. (2010). First report of western bean cutworm (*Striacosta albicosta*) in Pennsylvania. Crop Manag..

[11] Ingerson-Mahar J. (2012). Western bean cutworm found in New Jersey. Plant Pest Advis..

[12] Sánchez-Peña S.R., Torres-Acosta R.I., Camacho-Ponce D. (2016). The second report of the western bean cutworm, *Striacosta albicosta* (Smith) (Lepidoptera: Noctuidae) as a dominant corn pest in Mexico. Proc. Entomol. Soc. Wash..

[13] Chludzinski M.M. (2013). Biology and Management of Western Bean Cutworm (*Striacosta albicosta* Smith) in Michigan Dry Beans (*Phaseolus vulgaris* L.). Master’s Thesis.

[14] Paula-Moraes S.V., Hunt T.E., Wright R.J., Hein G.I., Blankenship E.E. (2012). On-plant movement and feeding of western bean cutworm (Lepidoptera: Noctuidae) early instars on corn. Environ. Entomol..

[15] Western Bean Cutworm in Corn and Dry Beans. http://extensionpublications.unl.edu/assets/pdf/g2013.pdf.

[16] Paula-Moraes S.V., Hunt T.E., Wright R.J., Hein G.I., Blankenship E.E. (2013). Western bean cutworm survival and the development of economic injury levels and economic thresholds in field corn. J. Econ. Entomol..

[17] Archibald W.R., Bradshaw J.D., Golick D.A., Wright R.J., Peterson J.A. (2017). Nebraska growers’ and crop consultants’ knowledge and implementation of Integrated Pest Management of western bean cutworm. J. Integr. Pest Manag..

[18] Eichenseer H., Strohbehn R., Burks J. (2008). Frequency and severity of western bean cutworm (Lepidoptera: Noctuidae) ear damage in transgenic corn hybrids expressing different *Bacillus thuringiensis* Cry toxins. J. Econ. Entomol..

[19] Ostrem J.S., Pan Z., Flexner J.L., Owens E., Binning R., Higgins L.S. (2016). Monitoring susceptibility of western bean cutworm (Lepidoptera: Noctuidae) field populations to *Bacillus thuringiensis* Cry1F protein. J. Econ. Entomol..

[20] Smith J.L., Lepping M.D., Rule D.M., Farhan Y., Schaafsma A.W. (2017). Evidence for field-evolved resistance of *Striacosta albicosta* (Lepidoptera: Noctuidae) to Cry1F *Bacillus thuringiensis* protein and transgenic corn hybrids in Ontario, Canada. J. Econ. Entomol..

[21] Bowers E., Hellmich R., Munkvold G. (2014). Comparison of fumonisin contamination using HPLC and ELISA methods in *Bt* and near-isogenic maize hybrids infested with European corn borer or western bean cutworm. J. Agric. Food Chem..

[22] Storer N.P. (1999). The Corn Earworm, *Bt* Transgenic Corn and *Bt*-Resistance Evolution in a Mixed Cropping System. Ph.D. Thesis.

[23] Horner T.A., Dively G.P., Herbert D.A. (2003). Development, survival and fitness performance of *Helicoverpa zea* (Lepidoptera: Noctuidae) in MON810 *Bt* field corn. J. Econ. Entomol..

[24] Bilbo T.R., Reay-Jones F.P., Reisig D.D., Musser F.R., Greene J.K. (2018). Effects of *Bt* corn on the development and fecundity of corn earworm (Lepidoptera: Noctuidae). J. Econ. Entomol..

[25] Liu Y.B., Tabashnik B.E., Dennehy T.J., Patin A.L., Bartlett A.C. (1999). Development time and resistance to *Bt* crops. Nature.

[26] Gould F. (1998). Sustainability of transgenic insecticidal cultivars: Integrating pest genetics and ecology. Annu. Rev. Entomol..

[27] Bates S.L., Zhao J.Z., Roush R.T., Shelton A.M. (2005). Insect resistance management in GM crops: Past, present and future. Nat. Biotechnol..

[28] Vélez A.M., Alves A.P., Blankenship E.E., Siegfried B.D. (2016). Effect of Cry1F maize on the behavior of susceptible and resistant *Spodoptera frugiperda* and *Ostrinia nubilalis*. Entomol. Exp. Appl..

[29] Montezano D.G., Hunt T.E., Specht A., Luz P.C., Peterson A.J. (2019). Life-history parameters of *Striacosta albicosta* (Lepidoptera: Noctuidae) under laboratory conditions. J. Insect Sci..

[30] Montezano D.G., Specht A., Bortolin T.M., Fronza E., Sosa-Gomez D.R., Roque-Specht V.F., Pezzi P.P., Luz P.C., Barros N.M. (2013). Immature stages of *Spodoptera albula* (Walker) (Lepidoptera: Noctuidae): Developmental parameters & host plants. Anais Acad. Bras. Ciênc..

[31] Angulo A.O., Jana S. (1982). La pupa de *Spodoptera* Guenée, 1852, en el norte de Chile (Lepidoptera: Noctudae). Agricultura Técnica.

[32] Efron B. (1998). Logistic regression, survival analysis, and the Kaplan-Meier curve. J. Am. Stat. Assoc..

[33] War A.R., Paulraj M.G., Ahmad T., Buhroo A.A., Hussain B., Ignacimuthu S., Sharma H.C. (2012). Mechanisms of plant defense against insect herbivores. Plant Signal. Behav..

[34] Blickenstaff C.C. (1979). History and Biology of the Western Bean Cutworm in Southern Idaho, 1942–1977.

[35] Antonelli A.L. (1974). Resistance of *Phaseolus vulgaris* Cultivars to Western Bean Cutworm, *Loxagrotis albicosta* (Smith), with Notes on the Bionomics and Culture of the Cutworm. Ph.D. Thesis.

[36] Doyle M.S. (1994). Pheromone Trapping and Laboratory Rearing of Western Bean Cutworm (Lepidoptera: Noctuidae). Master’s Thesis.

[37] Dyer J., Lewis L., Sappington T., Coates B., Abel C., Bidne K., Gunnarson B., Hellmich R. (2013). Western Bean Cutworm Laboratory Rearing Manual.

[38] DiFonzo C.D. (2010). Managing Western Bean Cutworm in Dry Beans.

[39] Esperk T., Tammaru T., Nylin S. (2007). Intraspecific variability in number of larval instars in insects. J. Econ. Entomol..

[40] Nijhout H.F. (1975). A threshold size for metamorphosis in the tobacco hornworm, *Manduca sexta*. Biol. Bull..

[41] Milks M.L., Burnstyn I., Myers J.W. (1998). Influence of larval age on the lethal and sublethal effects of the nucleopolyhedrovirus of *Trichoplusia ni* in the cabbage looper. Biol. Control..

[42] Reisig D.D., Reay-Jones P.P. (2015). Inhibition of *Helicoverpa zea* (Lepidoptera: Noctuidae) growth by transgenic corn expressing *Bt* toxins and development of resistance to Cry1Ab. Environ. Entomol..

[43] Huang F., Buschman L.L., Higgins R.A. (1999). Susceptibility of different instars of European corn borer (Lepidoptera: Crambidae) to diet containing *Bacillus thuringiensis*. Biol. Microb. Control..

[44] Farhan Y., Smith J.L., Schaafsma A.W. (2017). Baseline susceptibility of *Striacosta albicosta* (Lepidoptera: Noctuidae) in Ontario, Canada to Vip3A *Bacillus thuringiensis* protein. J. Econ. Entomol..

[45] Pannuti L.E.R., Paula-Moraes S.V., Hunt T.E., Baldin E.L.L., Dana L., Malaquias J.V. (2016). Plant-to-plant movement of *Striacosta albicosta* (Lepidoptera: Noctuidae) and *Spodoptera frugiperda* (Lepidoptera: Noctuidae) in maize (*Zea mays*). J. Econ. Entomol..

